# Advancing mHealth Research in Low-Resource Settings: Young Women’s Insights and Implementation Challenges with Wearable Smartwatch Devices in Uganda

**DOI:** 10.3390/s24175591

**Published:** 2024-08-29

**Authors:** Monica H. Swahn, Kevin B. Gittner, Matthew J. Lyons, Karen Nielsen, Kate Mobley, Rachel Culbreth, Jane Palmier, Natalie E. Johnson, Michael Matte, Anna Nabulya

**Affiliations:** 1 Wellstar College of Health and Human Services, Kennesaw State University, Kennesaw, GA 30144, USA; kgittner@kennesaw.edu (K.B.G.); mlyons30@kennesaw.edu (M.J.L.); jpalmier@kennesaw.edu (J.P.); 2 College of Computing and Software Engineering, Kennesaw State University, Kennesaw, GA 30144, USA; kmoble23@students.kennesaw.edu; 3 School of Public Health, Georgia State University, Atlanta, GA 30303, USA; knielsen4@gsu.edu; 4 American College of Medical Toxicology, Phoenix, AZ 85028, USA; rachel.culbreth@acmt.net; 5 Division of Clinical Epidemiology, Department of Clinical Research, University Hospital and University of Basel, 4051 Basel, Switzerland; nat.johnson@unibas.ch; 6 Uganda Youth Development Link, Kampala P.O. Box 12659, Uganda; mattemichael18@gmail.com (M.M.); nkanna.kavuma@gmail.com (A.N.)

**Keywords:** mHealth technology, digital health, wearable sensors, wearable devices, low- and middle-income countries, Uganda, Africa

## Abstract

In many regions globally, including low-resource settings, there is a growing trend towards using mHealth technology, such as wearable sensors, to enhance health behaviors and outcomes. However, adoption of such devices in research conducted in low-resource settings lags behind use in high-resource areas. Moreover, there is a scarcity of research that specifically examines the user experience, readiness for and challenges of integrating wearable sensors into health research and community interventions in low-resource settings specifically. This study summarizes the reactions and experiences of young women (N = 57), ages 18 to 24 years, living in poverty in Kampala, Uganda, who wore Garmin vívoactive 3 smartwatches for five days for a research project. Data collected from the Garmins included participant location, sleep, and heart rate. Through six focus group discussions, we gathered insights about the participants’ experiences and perceptions of the wearable devices. Overall, the wearable devices were met with great interest and enthusiasm by participants. The findings were organized across 10 domains to highlight reactions and experiences pertaining to device settings, challenges encountered with the device, reports of discomfort/comfort, satisfaction, changes in daily activities, changes to sleep, speculative device usage, community reactions, community dynamics and curiosity, and general device comfort. The study sheds light on the introduction of new technology in a low-resource setting and also on the complex interplay between technology and culture in Kampala’s slums. We also learned some insights into how wearable devices and perceptions may influence behaviors and social dynamics. These practical insights are shared to benefit future research and applications by health practitioners and clinicians to advance and enhance the implementation and effectiveness of wearable devices in similar contexts and populations. These insights and user experiences, if incorporated, may enhance device acceptance and data quality for those conducting research in similar settings or seeking to address population-specific needs and health issues.

## 1. Introduction

There has been a dramatic increase in the adoption and application of mobile health (mHealth) technology, specifically the use of wearable sensors and trackers, in many parts of the world, as a strategy for improving and measuring health behaviors and outcomes. In fact, mHealth, the innovative use of mobile health technology to enhance and support health objectives, has gained momentum also in low-resource settings [1,2]. The increasing uptake and availability of mobile phones, smartwatches and other wearable sensors, even in resource-constrained areas, has made mHealth an attractive option for monitoring health behaviors, health outcomes and supporting healthcare services [3]. Despite mHealth’s potential to enhance health outcomes, adoption in low-resource settings appears to be hindered by limited research focusing on the application and use of these devices among researchers and practitioners aiming to improve outcomes [4] or among participants in community settings. However, these uses of mHealth devices may be of significant benefit to countries across Sub-Saharan Africa (SSA) where the World Health Organization indicates a severe shortage of healthcare workers [5]. As such, mHealth technologies hold promise for addressing the lack of healthcare professionals and high disease burden in SSA by facilitating low-cost and scalable remote monitoring and tracking [6] and also advancing and accelerating health research. Unfortunately, the adoption of mHealth remains fragmented in low- and middle-income countries (LMICs), underscoring the need for targeted research exploring device implementation that can mitigate potential barriers and enhance uptake in technology use in addressing urgent health needs.

A recent review of mHealth usage in SSA found that only 2% of the studies examined used wearable technology [6]. Also, in that review, of the 48 countries in SSA, only 21 countries had implemented mHealth strategies into their healthcare systems at the time of that analysis [6]. The limited inclusion of mHealth in low-resource settings highlights the opportunity to borrow strategies and lessons learned from high-resource settings to amplify and leverage technology in health research and clinical practice to increase interest and capacity where uptake so far remains minimal [7,8]. Limited financial resources are clear barriers to the availability and accessibility of new technology in many of these settings. So far, mHealth strategies primarily through the use of wearable technology but also other tools, across LMIC settings have primarily addressed chronic disease management and monitoring for conditions such as diabetes, stroke, and cardiovascular disease [9]. These approaches have also been applied to prevention and intervention efforts in LMICs related to promoting a healthy diet and physical activity, supporting sexual and reproductive health, encouraging medication adherence, and support for HIV care [10]. Intriguingly, the innovative adoption of mHealth in new settings and contexts may support examining a range of other health concerns. For example, prior work has evaluated the potential of a wearable sensor to detect limb motion for the purpose of stroke evaluation and rehabilitation in SSA [11], the use of wearable electrocardiography (ECG), electroencephalography (EEG), and proximity sensors, in conjunction with surveys to collect data on child development in Malawi [12], the use of wearables to examine the effect of extreme weather on population health in Burkina Faso [7,13] and heat exposure in Kenya [14], to study community exercise in Ghana [15] and the use of voice messages and tablets to improve hypertension and diabetes self-management in Cambodia [16]. In this context of innovation and new applications, it is clear that there are tremendous opportunities for mHealth applications across SSA with appropriate capacity building.

Research indicates that mHealth interventions targeting health outcomes’ improvement in low-resource settings show promise for impact and a potentially large return on investment, emphasizing the importance of facilitating adoption and system implementation for scalability and sustainability for wide acceptance by healthcare workers [17,18,19]. While there is a positive trend towards the use of mHealth innovations in low-resource settings, there are concerns about sustainability and ethics considerations, particularly regarding patient or participant autonomy, safety, and justice [20,21]. However, it is important to note that comparisons of user experiences to high-income countries are not very helpful and relevant given the widely accepted and prevalent use of consumer-grade wearable technology [17].

The use of mHealth devices in low-resource settings may also be hindered by cultural factors including beliefs, values, or customs providing high device non-compliance when used for health monitoring outside of clinical settings [22] which is why assessing participant perspectives across contexts is very important and why we made that central to this paper. Logistical challenges, particularly in low resource settings, such as device management and internet connectivity issues can impact data collection, quality, usage [8], and the ability to keep the devices charged if electricity is not available [23]. There are also possible obstacles to using wearable mHealth technology in low-resource settings related to mobile phone use as many wearable sensors need to be paired with a smartphone. Lack of acceptance by patients and health care professionals and lack of necessary institutional frameworks for the use of technology may also be barriers to clinical use [24]. A key obstacle to the wider acceptance of wearable technology across Africa is related to the low rate of internet usage, with an estimated 22% of the population being connected to the internet [9]. However, research on the user experience of wearables in low-resource settings is emerging [7,15,16,23] and is urgently needed to grow capacity and to inform implementation strategies and data quality.

Research indicates that mHealth technologies can streamline and enhance the delivery of public health interventions. However, challenges such as the need for technologies to be adaptable to local context, the inherent complexity of different environments, and the lack of widespread digital infrastructure can obstruct their effective implementation [25,26]. Challenges like loss of trust among users and inadequate feedback mechanisms further complicate the deployment and integration of mHealth solutions. Additionally, the usability of mHealth technology such as wearable sensors in low-resource settings is not well understood due to the limited research to date on the user experience and findings specific to these environments [27]. This underscores the need for more research to address cultural, environmental, and implementation challenges to assist future researchers enhance understanding of mHealth device utilization in LMICs.

An intriguing aspect of using mHealth technology such as wearable sensors is the ability to track peoples’ activity in their natural contexts as they go about their daily lives, which offers innovative opportunities for understanding and contextualizing health-related behaviors and outcomes [28]. Wearable sensors can allow for continuous collection of heart rate, sleep, and movement [29,30]. As such, wearable sensors open the possibilities for many new options and scalable projects in community and clinical research and practice such as the ability to monitor patient compliance, monitor chronic disease, and detect issues earlier [30]. The use of wearable technology in low-resource settings can also enhance data collection in communities and improve communication between healthcare providers and patients [23]. While wearable sensors have shown success in clinical settings, their application in community environments requires adaptation and further exploration to ensure their effectiveness outside of clinical settings while still improving health outcomes and communication.

Overall, mHealth has the potential to significantly impact health research, healthcare delivery and medical resource allocation in low-resource settings, offering opportunities to improve access, quality, and adherence to care [30,31]. To be effective for health monitoring, wearable sensors need to be accepted by a population, but surprisingly few studies report on the feasibility and acceptability of wearable devices, specifically in low-resource and research contexts [8,23]. As such, addressing challenges related to implementation, culture, ethical considerations, and usability is crucial to realizing the full benefits of mHealth in improving healthcare outcomes in resource-constrained environments.

The purpose of this study is to examine the user experience and the methodological considerations learned from six focus groups exploring wearable device implementation. The specific goal was to capture participants’ user experiences after being outfitted with a consumer-grade fitness tracker in a setting where these wearable devices are not yet widely adopted. We collected this information to support the development and implementation of a larger prospective cohort study protocol using wearable devices. The TOPOWA project (TOPOWA means to not give up in Luganda, a local language spoken in Uganda) assesses the mechanistic pathways of mental illness and evaluates a community-based intervention to uplift and empower young women living in poverty. As part of this pilot study, which preceded the launch of the cohort study, we report the qualitative findings of the reactions, perceptions and experiences of using the wearable trackers by young women living in poverty in Kampala, Uganda. The findings from this observational study of wearables and in-depth assessment of the user experience can inform and facilitate the uptake of wearable sensors in research and clinical health settings across Uganda and in similar low-resource settings to improve health and healthcare.

## 2. Materials and Methods

This study describes focus group data collected as part of a 2-phase pilot study that explored the implementation of outfitting women with wearable Garmin vívoactive 3 smartwatches (Garmin, Ltd., Olathe, KS, USA) in Kampala, Uganda. A total of 60 women were enrolled on two pilot studies (N = 30 for each pilot) which involved wearing the Garmins and participating in a focus group and completing a short survey. Among those 59 women participants (ages 18–24) who consented and proceeded to participate in the focus groups, recorded data from 57 women were obtained from February to May of 2023. Women were recruited by our community implementing partner, the Uganda Youth Development Link (UYDEL), from three study sites in Kampala representing the informal settlements or slums in Banda, Bwaise, and Makindye. Inclusion to the study was restricted to women between the ages of 18 to 24 years and residing within one of the three study sites. We conducted focus groups separately with women at each of the three study sites.

Data were collected in a two-phase pilot study. The young women were outfitted with Garmin smartwatch devices to track their location, sleep, activity, and heart rate for sleep and sensed activities for five days. Note that the data collection was passive and was only collected and analyzed after the Garmins had been returned. As such we consider that study component observational. The data collected by the Garmins were not shared with participants in real-time. Subsequently to wearing the Garmin, participants were asked to share their experiences with wearing the Garmins in a focus group format and also through an anonymous survey. The results of the first pilot and the first three focus groups were used to inform and enhance the procedures and training for the second pilot study. For the second pilot, additional information was included in the training of the research team and of participants. Participants were also provided with take-home instructional materials to explain the device and its functions. Moreover, an optional fabric covering was offered for any participants interested in concealing the wearable device while wearing it. In both face-to-face trainings of the participants, they were instructed to leave the device on, if possible, during the day and night, for five days. They were also told to not engage with the device or tamper with its settings. The goal of all six focus groups was to understand the user experiences with and perceptions of the wearable devices to adjust the protocol as needed to improve future data collection and data quality for the planned cohort study.

All focus group discussions were led by a bilingual, trained and experienced facilitator who served as a moderator and a bilingual transcriber who took the notes. Each group discussion commenced with the introduction of the moderators and participants of the study along with describing the context and purpose of the focus group discussions. Each participant selected a pseudonym to ensure privacy in transcribed notes and discussions. Focus group discussions were conducted in both English and Luganda, a local language in Uganda, as the research team wanted to accommodate all participants’ preferred languages. For analysis purposes, the Luganda portions of the discussions were translated to English by researchers proficient in both English and Luganda, with the English-translated focus group transcripts being used for the analysis.

All focus groups used the same six semi-structured prompts to guide the focus group discussion (See Table 1).

Please note, throughout this document, the Garmin smartwatches outfitted on participants will be interchangeably referred to as “devices”, “watches”, or “wearable devices” despite all being the same Garmin product primarily marketed as a wearable activity tracker. These specific Garmin’s were selected because they did not need to be paired with a smartphone and they also had the longest battery life of the available devices with similar features, at the time of the study implementation. It is important to note that these watches are not considered medical devices, although our findings could potentially extend to such tools used beyond their intended consumer application.

### 2.1. Participant Demographic Characteristics

Participants were recruited through a snowball sampling technique based on their engagement with our community implementing partner, the UYDEL., a community outreach and training organization. The study participants’ demographic information included 27 in the age group of 18–19 years and 33 from 20–24 years. A total of 17 young women had children, of which ten (10) had one child, five (5) had two children, and two (2) had three children. Moreover, 18 of the participants were co-habiting/living with their boyfriend/partner, twelve (12) were living with their biological parents, 26 with relatives, two (2) with friends, one (1) with her own children, and one (1) was living alone. Overall, their family size ranged from two to fifteen people. Four (4) of the participants received upper-level secondary schooling, 39 received lower-level secondary schooling, and 17 received primary-level schooling. Additionally, 46 of the participants identified as Christian and 14 as Muslim. Only 23% (14) of the participants were currently working. In terms of employable skills, 41 were undertaking hairdressing, 12 were undertaking tailoring, 1 baking, 2 leather bag making, 1 computer training, and 1 was undertaking baking and jewelry making at the UYDEL youth vocational training centers.

### 2.2. Data Analysis

Qualitative standards for reporting in this article follow suggested reporting guidelines [32]. An inductive phenomenological approach to data analysis was employed to identify themes across the data which tend to provide a broader, more expansive analysis of the entire body to extract themes for detailed interpretations [33,34]. All English-translated focus groups were uploaded to NVIVO (Lumivero, Denver, CO, USA) 14 for theme organization. The primary analyst used a postpositivist theory in framing the thematic analysis and bracketed prior assumptions to provide a perspective that is grounded in the focus group data [35]. Braun and Clarke’s six-step process was followed, including familiarization with data through repeated readings, development of initial codes, initial extraction of themes, revision and refinement of themes, naming of themes and detailed interpretation with quotes [35,36]. Intercoder agreement methods were then utilized to ensure the consistency of themes. One researcher independently coded all six transcripts, while a second researcher coded a randomly selected transcript. The primary themes identified by the secondary researcher were confirmed within the overall thematic structure of all six transcripts with a single discrepancy being rectified in the final analysis. An extensive audit trail was documented through this process through both NVIVO and researchers’ journals. Rich thick descriptions are used to provide a summary of themes with the entire research team involved in the wearable administration and data collection reviewing the results [37]. The broader team with extensive experience and research in Uganda and with wearables closely reviewed the theme and narrative to ensure cultural consistency of all results. This series of refinement aims to enhance the trustworthiness of the results by ensuring the credibility, transferability, and dependability of the findings [38].

The analyses are described across ten interrelated themes: 1. Device Settings and User Guidance; 2. Interrelated Challenges Encountered with the Device; 3. Reports of Discomfort and Comfort with Device; 4. Satisfaction with the Device; 5. Changes in Daily Activities due to Device Use; 6. Changes in Sleep Patterns due to Device Use; 7. Speculative l Device Usage; 8. Community Reactions Towards the Device; 9. Community Dynamics and Curiosity Surrounding the Device; 10. General Device Comfort by the end of the Study.

## 3. Results

The results below present findings across the ten overarching themes with embedded discussion and participant quotes. The results capture both positive and negative aspects of participants’ experiences as well as the challenges and benefits that the participants encountered related to the wearable device. This presentation of the findings aims to provide a comprehensive understanding of the participant*s’* experiences by including individual reflections on the devices and community perspectives as shared by the participants.

### 3.1. Device Display and User Guidance

A common discussion topic expressed by several participants surrounded device settings, including accidentally changing the settings and how to best follow the explicit instructions surrounding the device usage that had been provided. Some participants expressed apprehension regarding the belief of unintentionally interacting with the wearable device display which they thought could lead to functionality issues. “*As I was moving, the watch started changing. Like showing distance, pace. And I said, ‘I didn’t touch this watch, what is going on?*” Uncertainty regarding the implications of these observations on continued device operations was noted throughout the discussions. In one focus group from the first round of the pilot study, participants agreed that “*[no one] explained to us that it lights up at night*”. There was no clarification if participants were referring to the green optical sensor that faced the wrist or the illumination of the watch screen. Watch screens had been set to the lowest brightness setting possible. In the second round of the pilot study, several participants discussed having the ability to reach out to the research team for device clarification; “*the watch started saving, in the morning, and it took a long time. I called Madame*”. Additional setting issues were outlined regarding various device information such as locked screens, scrolling, text, various numbers, low battery warning, and misunderstanding device settings such as the 24-hour time measurement that was only noticed after the participant left the research facility (and were unfamiliar to many of the participants). Some participants returned to the research facility whereas others discussed reaching out to the “madame,” the dedicated point of contact for participants, for clarification on how to proceed. Having a dedicated point of contact provided a reliable resource to reach out to with any issues or questions participants encountered during the study. These reflections and observations regarding device settings highlight the need for very clear instructions, reassurance, and a participant point of contact regarding the device’s robustness against accidental inputs and to answer participant questions.

### 3.2. Interrelated Challenges Encountered with the Device

Women’s experiences wearing the devices were marked by several observations that were often reported in combination. More specifically, sleep challenges were commonly reported by participants because of the device’s green light (referring to the optical sensor on the back of the wearable device which is only seen when the wearable is taken off the wrist or a gap between the wrist and device is present) and frequent adjustments necessary during sleep routines. “*The watch was a bit difficult at night as you would wake up to see so much light on your bed and you wonder where it came from…and then when you wake up again it’s the same story*”. Other women discussed challenges associated with sleep posture shifting; “*I was sleeping with the watch, because we use the hand as pillows*”. Also, apprehension about water damage during daily tasks and work activities like washing was frequently reported. “*When I would wash clothes, or utensils, my hand would get affected by the water and change color which worried me*”. Some participants expressed a strong desire to remove or adjust the watch, but many feared it might affect study outcomes, so they refrained from doing so. A participant went as far as not removing the watch while getting an IV (Intravenous line) at the hospital. “*So, I was sick and I was going to the hospital to get a cannula [IV]…she was taking it off, and I said no, don’t untie here, place it here, or up here. But it never went off [my arm]*”. Moving around the community heightened some participants’ reflections over potential theft or loss of the device; “*The watch was most stressful, when walking from home and back, for I was worried of loosing it*”. While moving around in the community some women felt as though the visible light emitted from the devices made them a target:

*There is a way this watch lights up brightly when one walks a long distance. Even when someone is far away, they can see…this light, that is so unique, when it shows walked a distance, someone can easily tell that it is different from the other watches. So those youths have always been looking at me. One day, they even wanted to check me.*

Women participants who lived with a partner also reported partners’ scrutiny of the devices. “*[My boyfriend] then tried to twist my hand like this and he saw it lighting and you don’t even want to take it off*”. Overall, these comments highlight the complexities of introducing wearable health technology in low-resource settings, where cultural, social, and practical considerations play critical roles in participants’ experiences given the unfamiliarity with the devices and emerging technology. The interplay of sleep, activity, water exposure, device removal, and issues while walking or getting around, partner interaction, and lights, highlight the complex interconnected nature of participants’ experiences with these wearable devices.

### 3.3. Reports of Discomfort and Comfort with Device

A few participants experienced a range of discomforts while wearing the health tracking devices including itching, rashes, swelling, and in a few cases some physical pain. This discomfort varied widely among participants, with some reporting physical side effects like skin irritation and others feeling the device was a physical burden. Some comments were related to the fit of the watch band: “*I would lift it [up] when the hair is broken, and every time it broke, it would bring a rash which itched*”. Pain was mentioned in the context of sleep and as a reason for waking up when sleeping on the device. “*The first time I slept with the watch, I woke up feeling pain, it had pressed me*”. One woman reported uncertainty regarding pain in her arm being attributed to a side effect from wearing the device or from recently having received an IV in her arm. “*Now, when they put the watch on, at first it was very fine, but when I went back to the hospital, putting the medicine in the canula [IV], I started feeling pain. Now, I was like, is it the watch? Is it the medicine?*” However, some participants noted that discomfort diminished over time. “*The first day I wore the watch, it gave me a lot of discomfort because I wasn’t used to sleeping with it, but with time, I got used to it*”. A period of adjustment was often necessary for those wearing the device, even more so among women who never wore things on their wrists. “*The problem that I had with the watch is that I had never worn a watch before, since my childhood, not even bracelets*”.

Participants’ comfort while wearing the devices varied, with some reporting initial discomfort that eased over time. After being woken up during the first night of sleeping a participant noted “*I started to get used to it slowly by slowly…[overall] I was comfortable with the watch*”. Adjustments to how the device was worn, previous experiences with wrist adornments, and understanding the temporary nature of the study contributed to increased comfort levels. Many felt at ease due to the “*sleek and comfortable*” design, with reassurances about its water resistance and durability during the completion of daily activities. “*I didn’t have any problems with it because I was able to do what I wanted to do, whether washing, or showering. I would just go freely*”. Comfort was often achieved through acclimatization over the study’s five-day duration, also highlighting the adaptability of new health technologies in new environments relatively quickly.

There were also discussions regarding the physical impact of the devices. Participants attributed several experiences of illness to the device or mentioned that they believed the device may be compounding existing illnesses. One participant who visited the hospital and received treatment stated: “*I couldn’t explain what [was] the cause of the pain, I wondered whether it was the sickness, or the watch?*” A different woman discussed her thoughts that the device may be impacting her appetite. “*On the day I wore [the device]…I got home my appetite first disappeared. I bought bread, I didn’t have any taste of it. I bought “elindaazi”, I also failed to taste it. I then bought milk, the taste failed*”. Several participants also discussed their thoughts about the safety of the device and whether may be hurt by the device. “*I started seeing the sign on it, isn’t it going to hurt me? I stood there and was confused…[and] wasn’t feeling well*”. The new device complicated participants’ ability to differentiate if these experiences were due to being sick, were residual effects of prior sickness, or if they were side effects of wearing the device. Also, these observations suggest that participants may have vivid or suggestive imaginations of the features and effects of the devices.

### 3.4. Satisfaction with the Device

Several participants found several positive aspects through wearing the health devices, with reported appreciation surrounding benefits such as improved time management and planning, tracking of activity, and a newfound sense of responsibility:

*It helped me to manage time and program myself and work according to that timetable. It helped me know the time that I had gone to bed. I’d see the time I had taken while sleeping, the time I would wake up, or know the time I had spent in a particular place.*

One woman noted “*when I got the watch it made me feel responsible*”. Pride and perceived social status of the device conferred a status of distinguishment on participants. “*The swagger in itself. Most of us looked very smart with these watches. When someone sees the watch you are wearing is expensive, it adds value to you*”. Despite any negative perceptions, the sense of loss felt by some upon returning the device underscores its social status impact, “*[customers] admired it, I didn’t even want to return it, I didn’t want to bring it back*”. The positive association with the wearable devices that some participants discussed reflects an appreciation for their presence and potential unintended social benefits, even though the devices were used for passive data collection rather than active engagement with participants.

### 3.5. Changes in Daily Activities Due to Device Use

Some participants stated they changed or considered modifying their behavior when wearing the devices. This finding presents potential challenges for the accurate data collection and representation of usual activities. Participants reported that in some cases their behavior changes were spurred by issues related to privacy, potential theft and loss of the device, and social judgment. However, the impact of these devices on participants’ daily activities varied: a few participants increased their daily activities while others restricted as much travel outside their homes as possible while wearing the device. One participant stated, “*I walked a lot to see how the watch worked, how the light went off*”. Another participant reported ceasing all outdoor activities for the duration of the study. “*I didn’t want anything or someone to touch the watch or knock me out. Of course, I knew that if this watch got spoiled, they wouldn’t give me any money…[Interviewer: So you stopped your day-to-day activities?] Yes*”. Such modifications suggest that the data captured by the devices might not fully reflect the participants’ typical lifestyle, emphasizing the importance of considering the socio-cultural context in the deployment of health technology and training of the research team and participants prior to device deployment in the community. This complex interplay between the adoption of technology, personal experiences, and societal influences highlights the need to address the lived experiences and reflections of users to ensure successful technology integration and reliable data collection in both research and clinical settings.

### 3.6. Changes in Sleep Patterns Due to Device Use

Similar to changes in daily activities, the impact of wearing health-tracking devices on participants’ sleep patterns arose in all focus groups. “*I used to also sleep differently, because we had been told that we don’t have to touch it*”. Participants reported a range of sleep-related issues related to the devices, including fear of damaging devices during sleep, changes in sleeping positions to avoid pressing buttons or damaging the device, discomfort, difficulty falling asleep, and awakening of participants and other family members. The extent of disturbance varied across participants, but many participants mentioned poor sleep and being woken up, particularly because of the green light. One participant mentioned “*at night when I was asleep, okay I used not to wake up at night, but the watch usually wakes me up most of the time*”. These quotes suggest issues that need to be addressed when seeking to collect accurate sleep data, as the devices may alter natural sleep behaviors. This feedback highlights the need for incorporating wearable health technologies that minimize the impact on sleep quality to ensure the collection of reliable data.

### 3.7. Speculative Device Usage

The use of wearable health tracking devices prompted reactions by both participants and in their reports also community members who were not familiar with these types of devices or technology. Due to the lack of familiarity with these devices, there were many speculated purposes and features of these devices that participants discussed. Some of these speculative uses were envisioned by participants while many were raised through participants’ reports of community questioning and discussion when encountering family, friends, and others in the community while wearing the device. Gaining an understanding of these reported speculative uses of the devices will serve to prepare researchers and healthcare professionals for common misconceptions that can be proactively addressed. Some speculative use was grounded in participants’ experiences regarding the device lights and screen notifications encountered during use. One of the most common reactions to the device was disbelief in it being waterproof. This speculation was often quickly dispelled with participants testing its capacity; “*we poured a lot of water in the saucepan…then we knew it was waterproof*”.

Familiarity with the devices was limited to what was shared with participants when they were outfitted with the device by the research team. While participants were informed that these wearable devices only recorded participants’ heart rate, physical activity, GPS location, and sleep; participants and community members were often skeptical, according to participants, that additional information or audio not described by the research team may also be collected. Community members “*[said] I was being recorded”* and *“everything you say goes on the watch*”. Others were “*suspicious that the watch could capture their voices so, before they spoke*”. Participants also discussed how “*this is a phone not a watch*” and “*I was carrying a computer on my hand*”, further suggesting that the devices may be recording conversations. While some wearable devices can potentially be used as a phone if properly connected and paired with a smartphone, the devices in our study were not paired with smartphones. Several community members also mentioned video recording, saying “*that the watch is a camera and it’s recording them*”, These comments focused on privacy and intimacy in particular: “*They said, are you going to take a bath while putting on a knicker because the camera is on*”, and discussed with friends that “*intimacy issues were hard for your partner because they feared they might be getting recorded*”. Others discussed the potential consequences of activities being recorded and reevaluated their behavior while wearing the devices. “*My son is naughty and sometimes you want to slap him, then you remember you have the watch, someone might find out*”. While the devices were capturing and storing location data for later analysis, some participants believed or told community members that the device transmitted data, for example when discouraging potential theft: “*I told them that no one could steal it. That he or she would be traced*”. Some of these speculations regarding their activities being recorded presented an intrusion into personal activities that were perceived to be somewhat uncomfortable, as reported by many participants.

Speculative use of the devices also intersected with Ugandan cultural norms resulting in conspiratorial and supernatural device usage. One community member “*even said that “yii!” you brought the Illuminati*”, with the concern of the Illuminati being present mentioned by several participants. (References to Illuminati typically represent a mysterious secret order of those claiming scientific and or religious enlightenment.) Some participants also mentioned cultural traditions, such as “*the watch it is for witchcraft*”. Some women had also discussed and speculated how the devices would actively regulate their menstrual cycles; “*So when [the device] displayed 10 and I remember it was the 10th day…When I went back home, it happened*”. These perceptions or issues appear to be rooted in Ugandan traditions, with no documented justification for these fears beyond speculation from the community. Participants discussed other very imaginative properties of the devices and suggested that they “*can make us invisible…[where] you can go to another world*”, “*could torch other items*” and “*kill us at night in a drone*”. Others encountered community members and family that expressed imaginative device features such as the devices exploding; “*that’s the bomb…now you went and brought your bombs here*”. Some of these speculations or fears regarding physical harm may be based on experiences that some older community members have in recalling the Uganda civil war or perhaps current conflicts in neighboring countries. The speculation also intersected with potential cultural traditions and postcolonial fears in Uganda: “*whites give those items, and I was afraid because I have white stuff*”. Also, perceptions regarding the spreading of disease and drug use arose in the context of racial and American mistrust. As one community member stated, according to a participant, “*have you ever seen when people are in America but when then they get your body and inject it with drugs?*” and another speculated device features because of the association with an American-funded study “*infecting them because the whites spread diseases*”. These comments collectively also indicate very low levels of health literacy which may adversely impact other health topics and needs which may be more proactively addressed in future projects.

Finally, other speculative implications, albeit rare, of study participation raised by community members spanned kidnapping, medical procedures, and death. Discussion surrounding mistrust of the research team and device provided that the continued device usage would lead to researchers “*taking you underground*” and that “*you will know that they have come to kidnap [you]*”. Moreover, comments by community members, as reported by participants, that “*they will sacrifice you one day*” or “*you’re going to die*” may be seemingly alarming, but should be viewed within the context and long history of witchcraft, witch doctors and animal sacrifices which are still commonly endorsed and practiced by some in Uganda. Those unfamiliar with Uganda, or similar settings, may not be aware of these longstanding and historical traditions that are reflected in these interactions. In contrast, some participants also engaged in the speculative representation of the devices using traditional culture, movie characters, and dramatization to joke with community members. “*I told them that this is powerful, you cannot handle it*”. Making light of wearing the device through these conversations helped some participants mitigate any stress they may have had while wearing the devices.

### 3.8. Community Reactions towards the Device

The highly imaginative and very speculative uses described above provide context regarding perceptions of these unknown devices. Given the uncommon nature of these devices in low-resource Kampala slums, community members, as reported by the participants, had many things to say regarding the devices beyond speculated uses. Communities’ perspectives on wearable devices were continually mentioned throughout the focus groups. Community members included families, friends, strangers, business associates and customers, and leaders within the community that participants encountered and with whom they interacted. Many of these perspectives were unsolicited as the community members had never seen a device like this before. Participants’ interactions with the community included various discussions and encounters, highlighting both positive and negative reactions from community members. It is important to note that in the Ugandan slums’ patriarchal context, community and family members often felt compelled or entitled to comment on women’s behavior and attire. This context is crucial for understanding the participants’ experiences and interactions with community members. One participant was asked by a neighbor if “*I can buy it from you*”. Often it was family and friends in their local community who would ask to wear, share, or borrow the watch. “*My friend visited and came with a watch. He told me that we [should] switch watches. That I give him mine and he will give me his*”. Some people just wanted to inspect the device closer with a participant mentioning “*a friend every time I would pass by, would ask to look at the watch*”. Children’s curiosity was consistently discussed by participants as “*the kids at home always wanted to see it*”. Some participants had coworkers continue to bother them; “*even at work people kept asking me to give it to them. They told me to give them so they can also wear it*”. Outside of their known personal network, participants discussed “*other people that I didn’t know…[would say] you girl stop you have a nice watch let us share that watch and I would say [ahh] I am not sharing the watch*”. Participants also reported being approached by local authorities, as one noted, “*military men live near our area, they see me passing…and they say give us some watch and we also shine like you*”. The pervasive requests by both strangers and people the participants knew to share, borrow, and try on the devices represent a cultural phenomenon that many participants reported while wearing them. Generally, these community interactions were peaceful and friendly, but nonetheless, they highlighted that strangers, family, and known community members were noticing the devices that the participants were wearing.

Community interaction also reflected praise regarding the devices. Several participants discussed “*[getting] showered with praises for looking smart while walking around with [the device]*”. The praise participants received elicited responses from the participants. “*I also felt high…because people in our area rarely wear such watches*”. Community adoration of the wearable devices resulted in jovial nicknames being given to participants when interacting with their local community:

*That watch has made the neighbors to give me a nickname, now they call me big, because of the watch I wear. They have saying that I’m different and I’m big, they had never seen it, it is unique, and now every time I would come out of the house, they would say, big, big! …They said you were saying you sent for that watch from abroad and that it came in a special way.*

Another participant outlined “*even at home, I was given a promotion because of this watch. They have been calling me ‘rich woman’*”. Overall, the community noticed the unusual devices, often approaching and interacting with participants wearing the devices, highlighting their conspicuousness.

### 3.9. Community Dynamics and Curiosity Surrounding the Device

Since wearable devices were a rare sight in the Kampala slums, participants found themselves addressing numerous inquiries and interactions about these devices from community members. Beyond the requests to share, trade, and borrow the watch, they shared experiences of answering a wide range of questions posed by family members, neighbors, and even strangers. With their curiosity sparked, they expressed an eager desire to reach out and touch the device, drawn by its novelty, with some satisfaction after further visual inspection. “*When they wanted to touch it, I told them look there. Just watch and don’t touch*”. Children’s curiosity would lead them to try to grab the device. Beyond touching the device, some of the more commonly reported questions surrounded “*what I had on my hand?*...[And] *where did you get it*?” Many in the community wanted to gain a better understanding of the device. Participants reported various conjectures regarding where the device came from and who supplied participants with the device; from the United States, from their boyfriend, and so forth. During community questions, some participants outlined that the devices left people “*mesmerized, because they had never seen [these devices]*”.

Participants encountered a variety of inquiries about their devices, reflecting a strong community curiosity regarding their capabilities. Amidst all the initial inquiries participants faced, questions often delved into specific features of the device, such as whether it was waterproof, the ability to wear it comfortably during sleep, and its functionality, including whether it could take videos or was akin to a computer. Community members asked “*whether it is waterproof, how I manage to sleep with it? What its use is? I told them it was mine, but they kept asking me*”. Curiosity also extended to its lighting features and date display, with some even testing its capabilities by being asked to not remove it during activities like washing or showering, while community members watched participants complete the activity. Even though participants attempted to answer and describe the device, questions were continually raised by community members. Some of the probing questions from community members created confusion for the participants. For example, one participant recounted being asked “*Do you have a mirror on your hand? I said no, this is my watch*”. This widespread interest underscores the novelty and intrigue surrounding the device among peers and the community.

Participants were also asked if their personal relationships were affected by the device’s presence. A boyfriend noticed that a participant was avoiding him for the entire five days of wearing the device and stated “*so that’s the watch that’s been scaring you to come here to see me?*” Questions also centered around the constant wearing of the watch, with family and community members curious about its permanence on the participants’ wrists. “*My uncles were asking me, why the watch doesn’t leave your hand?*” Additionally, covering the device sparked further curiosity, leading to questions about why participants felt the need to hide it, “*when you [wear] the watch with the cloth, people start complaining asking ‘what are you covering’, they become inquisitive*”. These varied questions demonstrate a broad spectrum of intrigue and speculations from those around them.

Many participants navigated inquiries from family and friends about the origin of the devices, with questions often focusing on whether they had acquired them through lawful means or if they were stolen. In returning home with these new expensive-looking watches, a father quipped “*what is that? Who gave it to you? Are you a thief?*” Another friend interrogated the participant in disbelief; “*who lent you that watch because I don’t expect it to be yours*”. Many participants had to outline they were outfitted with the devices for a research study, yet in doing so the questions shifted to potential fears of theft that wearing the devices placed on participants. “*If [I] wanted to move alone [at night] from the house my mother could tell me ‘where are you going to?*’…*She would say this area there are too many thieves so don’t go*”. The general topic of devices being stolen was further impacted when participants were questioned and interacted with community members who are known delinquents or criminals:

*For me I stay near some of the bayaye [street thugs]. After two days, one of them came when it got his attention, and was like ‘now days you put on a watch, you even shower with it, and wash with it, we don’t understand you’ I told them to mind their business as I mind mine too. But they said, ‘if your neighbor is putting on an expensive thing, can’t you say something?’ You can feel scared that one day they will follow you and grab it away.*

“*What if someone stole it, what would happen*” was a common question asked by many in the community. Participants actively had to consider daily choices and activities based on this community question. Participants faced a range of other inquiries about the devices they wore, highlighting curiosity and speculations. These interactions underscored a mix of admiration, suspicion, and intrigue from friends, family, neighbors, community members, and strangers alike.

The optical sensor’s “*green lights under the watch attract people’s attention*” and became even more noticeable at night. One participant noted “*other people that I didn’t know…one night they saw me coming from work at the same time when I had the watch with light penetrating, they asked me, ‘Are there watches that shine like that, ahh let us see?’*” When participants were asked about the green lights, the experiences often were a segue into confrontation regarding the devices.

Family asking about the green light often occurred at night when the light was more noticeable. “*I sleep with my younger sister, now at night she would wake up and call me to remove my watch, because it was lighting*”. Family complaints and quarrels over the device were the most common issues reported by some participants wearing the device. “*[In] most cases, it was my family members that complained [about the device], not passersby*”. Some of the family infighting was due to some of the previously reported fictional use of the devices:

*I was taken to the hospital and a cannula was put on my hand. People at home then started to complain about the watch and asked me to remove it. They thought that the watch had caused the illness. I told them that my sickness was not in any way caused by the watch and that it did not have any problem, I was just sick. Actually, if the doctor had asked me to remove it, I would accept, but the doctor was okay with it. [My family] insisted and asked me to remove it, but I refused.*

Unfortunately, some family requests to remove the device were not limited to just verbal requests. One participant reported this interaction with her husband “*I did not want to take off the watch, but my husband and I were arguing, he got angry and pulled off my watch and threw it down*”. The participant’s husband then took the watch and refused to return it to the participant. Eventually, the husband relented and gave the watch back.

The speculative applications previously mentioned seemingly provided a basis for further criticism by some community members, who appeared to use these reasons as a foundation to critique those wearing the devices. Some of this criticism was through mocking participants for agreeing to wear the device. “*I was afraid to wear the watch…When my brother saw it, he started telling me that the one who gave me that watch seems to love me very much and seems to have some money. I was silent*”. As some of the community was unaware of how the device worked, several women were called naïve and stupid. “*Some guy called me naïve for finding me doing laundry with a watch on my hand*” with another community member exclaiming “*are you stupid, why are you washing with a watch?*” The speculative nature of the devices’ functions resulted in regular criticism and exclusion while wearing the devices. Community members in one participant’s neighborhood stated:

*‘go and explode from there, we don’t want to be involved in it, explode alone.’ They would say, ‘you’re looking for death. We just lost someone, and you want to be next’, and as they talked, I would not be allowed to be part of their conversations.*

Every focus group reiterated criticisms related to speculative device usage, emphasizing the apprehension some community members felt towards the devices due to their unfamiliarity and the uncertainty surrounding them.

Participants faced similar interactions in public encounters with friends, neighbors, and strangers. During their initial use of the watch at the study’s training facility, one participant reported feeling unsettled after being questioned about the device’s functions. “*[On the] first day, they saw us with watches, and asked us what they told you [about the devices]? The following day…they would look at you badly, or even respond rudely*”. The participant was puzzled by the questioning about the device, a topic her sister suggested she should ignore. While riding a taxi across town, one participant said she perceived that a stranger was looking at her arm with an odd expression. “*I thought he was going to take the watch, I was scared, I shouted in the taxi… he told the conductor that I was recording the people in the taxi. He called people and showed them the lights*”. In a separate incident, a neighbor disclosed that “*he ‘wanted’ me and the watch I had on my [arm].* Another participant had different type of interaction with a friend due to her unwillingness to lend her watch:

*[My friend] tried to remove the watch, I pulled away my hand and told him, the watch can only be removed after five days. After that he said he is no longer my friend…*

During the first round of the pilot study, two participants mentioned covering the device with any available cloth including a coat, bag, and handkerchief to reduce questions and community interactions; “*I would tie a handkerchief around my [arm]*”. Given these discussions, a fabric bracelet to cover the devices was provided to all participants in the second round of the pilot study. In using the cloth to conceal the device and avoid any community interactions “*when we met people, [my sister would] give me the cover to put on. As people didn’t have to touch the watch for it to bring other issues, as she didn’t want problems*”. Throughout all the focus group discussions, participants consistently highlighted the unpredictable nature of public comments and interest in the devices, debating the intensity of such interactions.

### 3.10. General Device Comfort by End of Study

Overall, the participants had positive experiences with the wearable devices by the end of the five-day pilot study along with the unintended positive experiences discussed earlier. While a few participants noted some negative issues while wearing the devices, others discussed general satisfaction and comfort with the devices. “*I was comfortable with the watch, reason being, you told us it would last for only five days which was not a problem*”. Also, several participants stated that they “*didn’t even want to return [the device]*”. Some of these women highlighted becoming so comfortable with the devices and the attention it brought some women that they discussed their disappointment in having to return the watch and felt stressed about the device return. “*I got stressed after the watch was taken away and I said, ‘What am I going to do now*?’” This suggests a high level of acceptance and integration into women’s daily lives. Despite some notable issues, wearable devices can be successfully incorporated into SSA communities with broad acceptance.

While most of the women reported being comfortable wearing the devices after five days a few also encountered and reported a mix of apprehension and unease when wearing the wearable devices. Their introspective reflections about the wearable devices were heavily influenced by community perceptions, interactions, and inquiries. These external factors amplified some of their internal fears and worries, highlighting the complex interplay between personal experiences and societal reactions to new technology. “*[From] the first time I wore this watch, I felt anxious, I was so scared*”. These emotions were deeply tied to perceptions about potential theft, societal judgment, and misunderstandings about the technology’s capabilities. Additionally, there was some anxiety about the devices recording participants’ sleep patterns and activity tracking, fueled by rumors and misconceptions. This led to a heightened sense of vulnerability among some participants which was echoed across the focus groups, illustrating the challenges of introducing wearable technology in low-resource settings where the uptake of mHealth and other forms of technology remains low, and health literacy also remains low.

## 4. Discussion

The goal of our focus group discussions was to understand the perceptions of wearable sensors in preparation for a cohort study of mental health outcomes among women in a low-resource setting where the community adoption of this technology remains very low. Overall, the introduction of wearable health devices in Kampala’s slums was met with great enthusiasm and interest by both participants and local researchers. That said, we noted important logistical issues and participant reflections in this research that we incorporated and addressed in our protocol development for the larger study. We share these insights and anticipate that these findings can be applicable to other projects and to researchers and clinicians interested in these mHealth tools. We developed a set of recommendations that outline pragmatic steps that reflect the findings that participants shared regarding their perceptions and reactions to these devices that we hope will prove to be invaluable for researchers undertaking similar initiatives utilizing wearable devices in similar low-resource settings. Also, for context, despite the concerns raised by some participants regarding theft, all devices used were returned during the pilot studies.

Based on the issues highlighted in the focus group studies, we developed a set of various methodological considerations that future community health researchers using mHealth technology in monitoring and evaluating health outcomes might consider. These considerations pertain to participant training, use of device covers, selection of the devices, and improving comfort while wearing the devices. However, it is also abundantly clear through the focus group discussions that there is a significant range of speculation about the new technology that may have different cultural contexts depending on a study’s population.

### 4.1. Device Covers

Several participants discussed feeling unsafe and uncertain with the wearable device. As such, the research team worked alongside participants to develop a solution to improve safety and emotional discomfort. We designed and provided a fabric wristwatch cover that participants could choose to use in the second pilot study. These were made from African print fabrics and denim in different colors and patterns and participants could choose among many to find a bracelet cover that suited them. Researchers working in similar contexts where devices are unfamiliar to the community may find that covers or other culturally appropriate clothing such as long-sleeved shirts, dresses, scarves, handkerchiefs, and/or shawls may increase the utilization of devices and decrease the attention and scrutiny from the community.

### 4.2. Device Selection

When incorporating devices into future studies or healthcare settings, consideration should be given to the brand, device sizes, and device options. The specific Garmin vívoactive 3 smartwatches were selected for this study based on price and available features. However, the watch face of the vívoactive 3 was relatively large when compared to the narrow wrist circumference and ulnar styloid of many young Ugandan women. The larger size of these devices may have contributed to some of the challenges and discomfort shared by the participants. Additionally, the availability of device settings such as optical sensor brightness and the ability to adjust screen brightness or turn off the screen may factor into decisions concerning device selection. Researchers should also consider breathable band materials and consider offering multiple options for participants to select for comfort as well as exploring hypoallergenic bands to prevent rashes and other irritations. Non-wrist-worn devices that are not visible in daily activities may also improve safety for future studies, including devices such as rings or patches.

### 4.3. Device Details

While outfitting wearable devices on participants, researchers can outline how the devices work and what the devices are collecting. Additionally, a point of contact should always be included for participant troubleshooting and to answer participant questions, as noted earlier. Many participants discussed having to reach out to a study team member during the study for clarification on device functionality, and participants were encouraged to reach out for questions or concerns throughout the five-day period. Based on feedback from the first pilot of the wearables, the study team provided training for participants and research assistants on addressing misconceptions about the watch to ensure that participants understood the device, and its purpose and that they felt safe. Participants received a short, written document to take home to provide a summary of the device options and data being collected with it, yet questions still emerged. Sharing more details regarding the data being collected can perhaps help to dispel reported speculative concerns regarding use while aiming to reduce some of the associated stress participants experienced. Offering a question session for all participants while outfitting them may also ensure that participants fully understand and feel comfortable wearing the devices. Participants might benefit from a direct internet link to product details if they wish to review more detailed documentation. Additionally, devices should be checked to ensure reminders and notifications are turned off when possible, and described to participants, to reduce participant concerns regarding unexpected notifications and vibrations and also to reduce tampering with the device settings.

### 4.4. Mobile Recharges

Participants experiencing low battery notifications highlight the challenges of using wearable devices in areas and communities with limited and unreliable electricity access. To counteract this, researchers should consider developing study protocols that confirm that device batteries are fully charged and that also test battery life before outfitting participants. Depending on study time frames and length of data collection periods, some researchers may also consider distributing portable and economical battery chargers that extend battery life for participants to ensure devices stay charged throughout the study. This may be important for study protocols that require more than 5 days of data collection (or the duration of battery length for selected devices). This strategy will require additional participant training to effectively use the chargers, but will improve the collection of complete data by the wearable device. While additional participant training might not be suitable for all research contexts, this strategy could be particularly beneficial for studies with small samples as the strategy will help increase data quality.

### 4.5. Reducing Device Irritation

As device irritation was the most reported issue among those sharing any discomfort from the devices, it was often exacerbated by water. As such, researchers might remind participants to dry their wrists after getting them wet as soon as possible. Participants should also be reminded to regularly clean the area around the watch and wrist. In hot and humid climate conditions, researchers might provide participants with small containers that contain powder designed for sensitive skin that can help absorb moisture and reduce friction between the skin and the device.

### 4.6. Dedicated Study Contact Person

Participants throughout the study discussed being able to reach out to a research team member with any issues. We highly recommend that projects like these have an identified point person, as has also been raised in previous studies and lessons learned using wearables [23]. While many researchers would intend to offer this support, our study results reiterate the importance of maintaining a dedicated point of contact for participants to reach out to with any issues or questions. We would encourage future researchers to take this one step further and actively remind participants to message or call regarding any fears or fictional speculative uses they may have while wearing devices as these may be mitigated early and strengthen data quality and protocol compliance.

### 4.7. Ethical and Safety Concerns

The focus group discussions underscored a range of issues by both participants and community members (as told by participants) reflecting a range of community interaction and curiosity stemming from wearing the devices in public for five days. Some women changed their behaviors and interactions, community members also interacted and probed for details about the devices and there was quite a range of speculation and imaginative use regarding the technology. Our study population reported that they experienced high engagement and questioning from community members and intimate partners. The pervasive requests by both strangers and people the participants knew to share, borrow, and try on the devices reflect on ‘mwoyo gwa ggwanga’ or ‘spirit of the tribe’, in Luganda [39]. This cultural phenomenon of generosity and the norm of sharing, even with strangers, is deeply ingrained in Ugandan culture and underscores the community’s inherent openness and connectedness. However, this cultural context may also interfere with privacy and ethical considerations in the study protocol implementation and needs to be factored into community and clinical settings where these devices may be used. The study team provided multiple safeguards to ensure that participants felt safe with the wearable devices, including improved education around misconceptions about the device, providing wearable wrist device covers to promote safety, and encouraging open and frequent communication between participants and the study team.

## 5. Conclusions

These findings underscore the importance of understanding the cultural context in deploying these devices and how data quality may be mitigated based on participants’ responses and behaviors. As we noted in previous work, gender-based violence remains quite prevalent among young women who live in the slums of Kampala, both within the context of intimate partners [40,41] and in the community in terms of physical fights (37%), being threatened with a weapon (28%) and being raped (30%) [42]. As such this context needs to be factored into the study design and participant training. Moreover, potential ethical concerns regarding safety and privacy also emerged for consideration given women’s lack of agency and empowerment in this patriarchal society [43,44]. These are important contextual issues for projects and interventions addressing women’s health needs. These are also important issues for future research to tackle and to develop strategies to mitigate their impact in order to improve women’s health.

Future research should also prioritize participant safety and address social and cultural challenges through comprehensive training and community engagement with devices. This includes selecting culturally appropriate devices with modifications being considered throughout incorporation to enhance comfort and acceptance. By acknowledging these dynamics and incorporating community input early such as this pilot study before launching a larger cohort study, researchers can foster a supportive environment and mitigate potential risks and challenges. Addressing these concerns is crucial to ensure the ethical and effective use of mHealth technologies, to which we offer some methodological considerations for future study design utilizing mHealth technology.

Additionally, while not a goal of the pilot studies, the findings from the focus group discussions also indicate very low levels of health literacy in the community given the discussions about the potential features of the devices and speculative outcomes of wearing them, which may need to be factored in for future health projects. Future research is also clearly needed to expand the knowledge base and adoption of new technology to strengthen the capacity for health research and clinical practice that leverages these tools, particularly in underserved populations.

In terms of the next steps for future research, it would be very important to continue to collect qualitative data about wearable devices and their experiences with these devices. Future research should also include iterative processes for wearable devices through a user experience framework. These iterative studies should thoroughly incorporate participant feedback in between each wearable data collection period, improving the participant’s experience through each cycle. Furthermore, studies should include an expansion of mHealth options for non-wrist-worn devices to optimize participant comfort, and safety, and reduce the participant burden. These studies may ultimately lead to scalable mHealth interventions in similar settings.

There are important limitations that should be considered when interpreting these findings. The relatively small sample size and specific population of young women living in poverty in this study may limit the generalizability of the findings to other populations or settings. Also, our findings reflect participants’ perceptions and reactions to these specific Garmin devices. Other wearable devices or sensors in different sizes, with different functionalities, battery lives, and other features may be perceived differently. In the focus group discussions, we also asked participants how they felt while they were moving around with the devices. Their responses reflect quite a bit of commentary and reactions from the social settings and community members. While we reported on these statements by community members, it is important to note that these were the participants’ reactions and perceptions to these conversations and interactions which included some very imaginative and speculative device features. There may be several biases that factored into their recollection and responses to these which we cannot disentangle. However, we think it is important for future researchers to consider these insights and perhaps also to engage community leaders and elders in understanding the social and cultural aspects of introducing new technology in close-knit communities where such technology has yet to be introduced and adopted. Finally, another potential limitation of this research is the primary analyst’s limited experience with sub-Saharan Africa, which, despite efforts to bracket assumptions and ground the analysis in focus group data, may have influenced the interpretation of the themes. However, several authors including the senior local author represent the cultural context, and she also provided oversight of the project implementation and has extensive experience with the study population. Moreover, Principal Investigator and lead author has conducted community-based research in Kampala for more than 15 years. All authors contributed to the interpretation of the themes and their context.

Despite these limitations, this study contributes to the scarce literature that describes the perceptions and reactions of young women after using wearable devices in Kampala slums. Our findings illustrate a landscape where community interest and speculation interweave with personal user experiences. Participants navigated a spectrum of reactions, from speculative and very imaginative use to practical reflections regarding their privacy, safety, and device functionality. These reflections shaped participants’ interactions within their communities and influenced their personal acceptance and adaptation to the technology. Despite initial anxieties by some participants, gradual acclimatization occurred, underscoring the devices’ perceived benefits and associated community status. However, the potential impact on daily routines discussed by participants signals a crucial need for tailored, culturally informed communication and study design strategies. As further detailed, these strategies should address misconceptions, enhance user comfort, and ensure wearable technology’s integration into daily life that is culturally sensitive and respects cultural boundaries. Ultimately, the study highlights the necessity of incorporating user-centric study designs with thorough contextual action in the deployment of health technologies, particularly in diverse, low-resource settings. Most importantly, the lessons learned from this study indicate that overall, the introduction of these wearable health devices sparked considerable enthusiasm and interest from participants, their communities and local researchers which is very promising for future projects and for the advancement in using these wearable devices in low-resource settings.

## Figures and Tables

**Table 1:** Focus Group Semi-Structured Prompts.

1. What were your overall experiences wearing the Garmin watches?
2. Did you remove the watch for any reason? If so, why and where?
3. How was your experience sleeping with the watch?
4. What was your experience travelling/walking with the watch?
5. What did you feel as you were walking through the community with your watch?
6. Were there any places that you felt unsafe while wearing your watch?

## Data Availability

These qualitative pilot focus group data can be requested from the lead author.
